# Oral health assessment in asthmatic children: An observational study

**DOI:** 10.6026/973206300221080

**Published:** 2026-02-28

**Authors:** Padmanidhi Agarwal, Ashi Chug, Lata Goyal, Kanav Jain, Aakash Gupta, Shailesh Kumar, Priyanka Mishra, Kamini Kiran

**Affiliations:** 1 Department of Dentistry, Dr. Ram Manohar Lohia Institute of Medical Sciences, Lucknow, India; 2 Department of Dentistry, All India Institute of Medical Sciences, Rishikesh, India; 3 Department of Dentistry, All India Institute of Medical Sciences, Bathinda, India; 4 Department of Conservative Dentistry and Endodontics, Chandra Dental College and Hospital, Safedabad, Lucknow, India; 5 Department of Dentistry, All India Institute of Medical Sciences, Gorakhpur, India; 6 Department of Conservative Dentistry and Endodontics, Institute of Dental Studies and Technology, Modinagar, India

**Keywords:** Caries, β _2_ agonist inhaler oral hygiene

## Abstract

Dental caries and bronchial asthma are related, but the effect of asthma severity and medication on oral health has conflicting evidence in the literature. Therefore, it is of interest to evaluate oral health indices in the outpatient Department of Dentistry, over a period of 2 years, in 1-17-year-olds undergoing treatment for asthma. Out of 168 children (115 male, 53 female), 13-17 year olds showed highest DMFT and Plaque index. Out of 77.5% of children with greater severity of asthma and duration >1yr, 61% children showed poor oral hygiene and signs of gingival inflammation. Children with chronic asthma a year ago had a higher mean deft/DMFT and also, significant proportional increase in mean deft/DMFT with age was observed. Oral hygiene was poorer and proportional to caries incidence and inhaler usage under-scoring importance of oral health maintenance after inhaler usage

## Background:

Bronchial asthma and dental caries both frequently affect children of all ages and their inter-personal relationship is a critical area of scientific investigation, but controversial at the same time [1]. Bronchial Asthma is a chronic inflammatory disease of the airway, in which there is increased sensitivity. It results in repeated wheezing, difficulty in breathing, chest constriction and coughing, primarily during night-time or early morning [2]. It is responsible for a major paediatric health burden worldwide. The focus of treatment of asthma involves symptomatic control of the bouts of wheezing and the frequent respiratory distress, that are a primary complaint. Management involves a cocktail of medications and inhalers according to type and severity of the disease [3]. Dental caries on the other hand is a non-communicable disease resulting commonly in children due to the frequent presence of favourable host-teeth surrounded by dietary fermentable monosaccharides in presence of lurking cariogenic bacteria, when left together for a substantial time. Risk of dental caries increases with lifestyle, other diseases and environmental factors that favourably affect any or all of these variables [4]. The oral cavity is negatively impacted by asthma with increased calculus formation, gingivitis, reduced salivary production, its altered composition and pH. The rise in likelihood of teeth cavities in asthmatic children treated with inhaled medications was frequently linked to a reduction in the rate of saliva production and elevated Lactobacilli and Streptococcus mutans levels [5]. Also, the desire to wash out the unpleasant taste of anti-asthmatic drugs and to counter oral dryness may lead to increased consumption of cariogenic drinks [6]. Poor oral hygiene in patients on inhaled corticosteroid therapy is also a concern as the corticosteroid molecules deposited in the oral cavity and pharynx after inhalation lead to local suppression of immunity which can further lead to various infections, predominantly fungal [7]. Bronchial Asthma, being a chronic respiratory disease, tends to focus more on medical care, whereas oral care is neglected, leading to poor oral hygiene and increased chances of caries [8]. Medications heighten the caries likelihood by decreasing saliva production and growth of cariogenic bacteria [9]. It is necessary to identify caries risk in children with asthma so that the required attention can be given to oral hygiene and special preventive dental health care programs can be formulated for them [10]. Therefore, it is of interest to assess the oral health and frequency of dental cavities in children diagnosed with bronchial asthma, by plaque index, deft (Decayed, extracted due to caries, filled teeth- deciduous) and DMFT (Decayed, Missing, Filled Teeth-permanent) Indices.

## Methodology:

This observational study was performed over a period of 2 years. All the patients reporting to the paediatric asthma clinic of the outpatient department (OPD) of Paediatrics, suffering from bronchial asthma, were screened. These patients were then examined and screened in the OPD of the Department of Dentistry at this major Tertiary care Centre of India. The study was approved by the Ethical Research Committee of the Institute (AIIMS/IEC/17/262) and conducted according to STROBE guidelines. The sample size was determined by convenience sampling. All the patients between 1 - 17 years diagnosed with bronchial asthma and undergoing treatment for at least three months, who reported within the 2-year span of the study were included in the study. Asthmatic children who were receiving oral care (tooth brushing by themselves or by a guardian) at least once a day and who had not received any intervention for caries prevention (in terms of fluoride application) were a part of this study. Those patients unwilling to participate or those suffering from other chronic diseases or other respiratory illnesses were excluded. Children's guardians were informed about the study. Written informed consent was obtained from the parent/ caregiver of each patient by signing the consent form. Their age, gender, history and duration of asthma (less than or more than a year), severity of asthma and treatment undertaken- whether there was any use of any medication/ inhaler, was recorded. The total number of primary and permanent teeth was noted and numbered using the FDI system. The severity of asthma was categorised as Mild, Moderate and Severe according to the clinical features of the Global Initiative for Asthma (GINA) Guidelines by the attending paediatrician. Each patient was examined with the Plaque Index to assess the oral hygiene of participating children. It was performed by a single examiner using a mouth mirror and a community periodontal index of treatment needs (CPITN) probe. It was categorised as good, fair and poor according to scores 0-0.9, 1-1.9 and 2.0 -3.0 respectively. The DMFT index (WHO criteria 1997) for evaluating dental decay was recorded using capital (DMF) and small letters (def) for permanent and primary teeth, respectively for decayed, missing and filled teeth. A mouth mirror and explorer on a dental chair with standard illumination was used by a single observer. For the final scores, DMF and def, all examination results were summed up accordingly. The comparison was made between various age groups and between the oral hygiene and caries status of children. The severity of asthma, caries and poor oral hygiene were also compared. Statistical analysis was done using Kruskal-Wallis test. SPSS software version 21.0 was used for analysis. All the comparisons were is made after proper diagnostics of the assumptions of normality and homogeneity of variances across groups to be compared. If these assumptions were found to be violated, an appropriate non-parametric test was used. A p-value of <=0.05 was found to be statistically significant.

## Results & Discussion:

In 2 years, 168 asthmatic children were screened, 68.5% male and 31.5% female. Gender disparity could be attributed to effect of sex hormones on lung development causing males to be afflicted with asthma more in childhood while females in adulthood [11]. 77.5% children had greater asthma severity and duration >1year, of which 61% showed poor oral hygiene and signs of gingival inflammation. 42.85% showed good oral hygiene, while 38.09% had poor hygiene (Table 1) which was directly proportional to caries prevalence. This can be attributed to changed microflora, with higher levels of Lactobacilli and S. mutans in asthmatics [12]. Children having chronic bronchial asthma (>1year) had significantly higher mean def and DMFT (Table 2). Caries indices were found to be high in asthmatics of all ages. This increase with age (p<0.05) highlights the susceptibility of asthmatics due to impaired enamel formation and subsequent defects due to diminished oxygen supply to active ameloblasts. Ameloblast inability for repair after injury after tooth development and eruption is due to their programmed cell death [3]. Potential mechanisms for higher caries prevalence include decreased salivary flow rate and salivary composition modifications in such individual, lower pH of plaque, high levels salivary S. mutans and regular intake of sweet laden drinks. Mouth breathing in asthmatics leads to dry mouth and want for frequent drinks [13]. DMFT was significantly higher in asthmatics (>1year) and as duration of asthma increased, there was a gradual decrease in salivary flow and oral p^H^. A positive correlation between illness duration and salivary levels of S. mutans in asthmatics exists [14]. The role of medications also contributes. No significant change in deft scores was observed with increased asthma duration probably due to shedding of the deciduous teeth as age advances. Across different oral hygiene levels, there was a difference in deft and DMFT (p<0.001). The mean rank of def scores was 115.27 for "Poor", 83.23 for "Fair" and 57.71 for "Good" oral hygiene. Poorer hygiene was associated with higher deft. Post-hoc comparison demonstrated increased level of hygiene with significant decrease in deft. The same was observed for DMFT. Significant differences were observed in distribution across different severity of asthma for deft, p=0.034 and DMFT p=0.007. A significant difference was observed only between mild and moderate levels of asthma with deft and DMFT (Table 3). Asthma severity was directly proportional to deft/DMFT scores, probably due to the different medications used by them- bronchodilator combinations (in mild cases), with inhaled glucocorticoids (in moderate cases) and with oral medications (in severe asthma). An inverse relationship between asthma severity and salivary flow rate has also been demonstrated [15]. The difference between moderate and severe asthma severity was significant, unlike between mild and severe. On exploring the severity association with def score within each level of oral hygiene- no difference in deft across severity was observed, indicating that only oral hygiene had statistically significant impact on deft.

Inverse results were obtained for DMFT with asthma severity. A significant difference for pairwise comparison for Mild-Moderate and Mild-Severe was noted. No significant change was observed in the DMFT with moderate to severe asthma (Table 3). Distribution of deft and DMFT scores across oral hygiene levels showed statistically significant differences (p<0.001) with direct proportionality (Table 4). Increased level of hygiene showed a significant decrease in deft and DMFT and severity/ frequency of caries was higher in asthmatics so it is crucial to consider them high-risk individuals and provide them with specialised care. To efficiently address and prevent caries, measures like gargling with sodium bicarbonate or utilising mouth rinses containing neutral sodium fluoride after using inhalers can be utilized [16]. When compared between inhaler users and non-users, both scores were significantly higher in users (p<0.001), indicating the associated risk (Table 4). This could be attributed to decreased salivary flow by the β_2_ agonists and corticosteroids, causing salivary and plaque p^H^ reduction and microbiome alteration, creating opportune environments for calculus and caries. Thus, harmful effects of medications are greater than the disease [16]. Some inhaled medications contain lactose. Prolonged presence of sugar on the surface of teeth results in prolonged acid production by cariogenic bacteria [3]. This tooth demineralisation has worse outcomes when combined with limited saliva, increased S. mutans and regular cariogenic food intake by asthmatic children. Oral bronchodilator use shows significantly higher occurrence of dental caries than those using dry powder or dose-metered inhalers [17]. A consultation with a pulmonologist is necessary for potential medication modification like dual-drug regimens or for guidance on strategies to minimise medicine-associated side effects. For caries prevention in asthmatic children prevention can mainly be achieved through public awareness and starting education on oral hygiene from a young age [1]. Dental check- ups should be mandatory. Toothbrushing with fluoridated toothpaste effectively reduces caries as it removes plaque from proximal surfaces. For individuals incapable of efficient plaque removal, regular in-office fluoride application as varnish can be used. Dental prophylaxis by clinicians is essential for good oral hygiene [17]. Strength of the study was novelty of discussing oral health indices for both deciduous and permanent teeth and comparing asthma severity and disease duration with these scores. Limitations included the choice of convenience sample, inability to record particulars of drugs (B_2_ agonists/ corticosteroids) used by patients, not controlling behavioural health patterns (dietary habits) and presence of covariates (socioeconomic status, etc.,) that could be potential confounding factors.

## Conclusion:

Oral health is compromised and proportional to caries in asthmatic children due to the disease as well as its medications, so preventive strategies should be timely implemented. More studies evaluating different modes/ doses of interventions can help formulate definite oral health maintenance plans. Preventive strategies should be timely implemented for asthmatics to be able to decrease oral health care burden in children affected by this chronic disease and obtain better outcomes.

## Funding:

No external source of funding

## Figures and Tables

**Table 1:** Demographic characteristics

**Age Group**	**Number of Children**	**Duration of Bronchial Asthma**		**Severity of Asthma**			**Gender**			**β_2_agonist inhaler**		**Oral Hygiene**		
		<1 year	>1 year	mild	moderate	severe	Male	Female	Yes	No	Poor	Fair	Good
1-4yr	17	15	2	9	6	2	13	4	6	11	5	3	9
5-8yr	67	18	49	10	8	49	51	16	27	40	20	10	37
9-12yr	55	4	51	1	3	51	29	26	28	27	29	12	14
13-17yr	29	0	29	0	0	29	22	7	17	12	10	7	12
Total	168	37	131	20	17	131	115	53	78	90	64	32	72

**Table 2:** Distribution of deft and DEFT in the various age groups and comparison with various factors

**Age Group**	**Number of Children**	**deft score**			**Mean deft**	**Average number of primary teeth**	**DMFT**			**Mean DMFT**	**Average number of permanent teeth**
In years	(n)	0-3	6-Apr	10-Jul	Score	(n)	0-3	6-Apr	10-Jul	Score	(n)
4-Jan	17	15	1	1	1.65±2.71	19.35	17	0	0	0	0
8-May	67	52	13	2	1.82±2.18	18.03	67	0	0	0.15±0.47	3.85
12-Sep	55	35	19	1	2.74±2.20	9.38	51	3	1	0.93 ± 1.33	16.31
13-17	29	29	0	0	0.03+0.18	3.38	16	7	6	3.41 ± 2.80	24.2
Total	168	103	33	4	2.0±2.20	-	151	10	7	1.0±1.84	-

**Table 3:** Pairwise Comparisons of deft, DMFT in different level of oral hygiene

**Criteria**	**Significance levels across different Levels**		
Comparison across Hygiene	Good-Fair	Good-Poor	Fair-Poor
deft	0.026	0	0.004
DMFT	0.396	0	0.125
Comparisons across asthma severity	Mild-Severe	Mild-Moderate	Severe-Moderate
deft	0.505	0.029	0.13
DMFT	0.008	0.029	>0.999
P-value of <=0.05 was found to
be statistically significant.
Significance values have been
adjusted by the Bonferroni correction
for multiple tests.

**Table 4:** Distribution of deft and DMFT across Oral Hygiene and Inhaler usage

**Parameter**	**Level**	**deft**		**p-value**	**DMFT**		**p-value**
		Median	IQR		Median	IQR	
Oral hygiene	Poor	3	5-Feb	<0.001	1	0-2	
	Fair	1	0-2		0	0-1	<0.001
	Good	0	0		0	0	
Inhaler Usage	No	0	0	<0.001	0	0-2	<0.001
	Yes	1	0-2		2	0-3	
A p value<=0.05
was considered statistically
significant and <0.001 was
found to be highly significant.
Abbreviations-
IQR- Interquartile range
